# Recognising Gender Discrimination: The Recognition–Normalisation Paradox in Italy and Lithuania

**DOI:** 10.3390/ijerph23070877

**Published:** 2026-07-05

**Authors:** Giulia Lausi

**Affiliations:** Faculty of Law, Vilnius University, Saulėtekio al. 9, 10221 Vilnius, Lithuania; giulia.lausi@tf.vu.lt

**Keywords:** gender discrimination, everyday sexism, predictive processing, normalisation, qualitative research, Italy, Lithuania, social cognition

## Abstract

**Highlights:**

**Public health relevance—How does this work relate to a public health issue?**

- Gender discrimination is a recognised determinant of psychological distress, mental health inequalities, and reduced wellbeing, particularly among women and gender-minority individuals.
- Despite its documented prevalence, discrimination often persists without being explicitly challenged, limiting the effectiveness of awareness-based public health strategies.

**Public health significance—Why is this work of significance to public health?**

- This study identifies the *recognition–normalisation paradox*: a cognitive and discursive mechanism through which individuals simultaneously acknowledge gender discrimination and attenuate its significance, preventing critical contestation.
- By integrating predictive processing theory with gender discrimination research, the study provides a novel theoretical framework that can be used to interpret why discrimination may endure even when recognised, with implications for how health-relevant social inequalities are reproduced at the cognitive level.

**Public health implications—What are the key implications or messages for practitioners, policy makers and/or researchers in public health?**

- Awareness-raising interventions alone are insufficient to challenge gender discrimination: effective programmes must also address the interpretative and normative frameworks through which discrimination is rendered socially expected and cognitively manageable.
- Cross-national evidence from Italy and Lithuania suggests that normalisation mechanisms are contextually configured, meaning that public health strategies should be adapted to the specific socio-historical gender arrangements of each context rather than applied uniformly.

**Abstract:**

Gender discrimination is a widespread phenomenon in contemporary societies; however, people often acknowledge its existence without challenging it. This study introduces the concept of the ‘recognition–normalisation paradox’, defined as the simultaneous recognition of gender discrimination and its mitigation through interpretative frameworks that render it socially acceptable, and proposes a theoretical framework based on predictive elaboration and social cognition to explain its cognitive underpinnings. Semi-structured interviews were conducted with 25 participants (14 in Italy, 11 in Lithuania) of both genders, analysed using an integrated qualitative–quantitative approach with ATLAS.ti (v. 26.0.1). The findings reveal that recognition and normalisation systematically coexist within the same narrative sequences. The cross-national analysis indicates that, whilst the paradox is structurally invariant in both contexts, its specific configuration differs: in Italy, normalisation operates predominantly through routinised relational and familial expectations, whereas in Lithuania it is achieved through discursive relativisation and contextual distancing. These findings challenge the view that normalisation reflects an absence of recognition, reframing it as a phenomenon that can be interpreted as a form of inferential processing. The implications for interventions promoting gender equality are discussed.

## 1. Introduction

Gender discrimination is widely acknowledged as a pervasive phenomenon of contemporary societies, which manifests itself not only in overt acts of exclusion, but even more so in subtle, often ambiguous, interactions that occur in everyday life [1,2]. A substantial body of psychological research has documented the prevalence of everyday sexism, micro-level inequalities, and the reproduction of gendered hierarchies through everyday social practices [3,4,5,6]. These studies have consistently shown that discrimination frequently operates in indirect and socially embedded forms, making it less visible and, consequently, less likely to be explicitly identified, labelled, and challenged by those who experience it. However, recognising gender discrimination is not a straightforward perceptual act. Rather than being immediately self-evident, many discriminatory experiences require active interpretative work [7,8]. Individuals must classify an interaction as unfair, gender-based, socially problematic, and structurally significant: this is a sequential and cognitively demanding process that takes place under conditions of ambiguity, making it contested or inconclusive: episodes may be experienced as contextually contingent or open to multiple plausible interpretations. As previous research has argued, the routinisation of inequality contributes to its normalisation, embedding discriminatory patterns within socially accepted practices and thereby making them seemingly invisible [3,9]. Yet, while existing research has extensively documented the pervasiveness of subtle and routinised forms of gender discrimination, comparatively less attention has been devoted to the cognitive and inferential processes through which such experiences are interpreted, recognised, or made socially unremarkable. This represents a critical analytical gap, particularly in understanding why individuals frequently articulate awareness of inequality while simultaneously minimising or normalising it.

### 1.1. Predictive Social Cognition and Gendered Social Priors

To address this gap, the present paper draws on insights from predictive processing and social cognition. Clark’s [10,11,12,13] influential account of the predictive brain proposes that perception and interpretation are not passive responses to external stimuli but rather the outcome of ongoing inferential processes guided by prior expectations. The brain operates as a hierarchical prediction machine, continuously generating models of the world and updating them in response to prediction error: the discrepancy between anticipated and incoming sensory or social information. Perception, on this account, is fundamentally constructive: individuals do not simply register reality but actively infer it through the continuous integration of top-down predictions with bottom-up sensory data. This framework gains on analytical significance when applied to social cognition. Hinton [14], in his examination of culture and implicit cognition, has argued that culturally sedimented schemas structure social perception in ways that largely escape reflective awareness. Culturally transmitted expectations are rooted in inferential patterns and thus determine what we perceive, how we classify it, and the resulting reactions that are elicited, even before a conscious evaluative process takes place. Applied to gender relations, this implies that individuals interpret social interactions through gendered priors: internalised expectations concerning gender roles, appropriate conduct, distributions of authority and power, and normative patterns of emotional expression and relational vulnerability [15]. These gendered priors operate across multiple dimensions of social cognition simultaneously. They determine what is perceived (is a comment considered sexist or does it go unnoticed?), how an interaction is interpreted (is that same comment a joke? a form of discrimination? something said “just for the sake of it”?), and the severity with which the incident is perceived (is it an isolated event and, therefore, irrelevant, or is it a symptom of a systemic pattern?) [16]. Recent work in predictive social cognition suggests that perception is not a passive process but is shaped by prior expectations and learned schemas that guide interpretation of social information [15,17]. Within this framework, the recognition of discrimination can be understood as an inferential process, in which individuals interpret ambiguous or familiar situations in light of pre-existing beliefs about what constitutes “normal” social interaction [15,18]. From this perspective, the concept of predictive recognition refers to the idea that discrimination may be simultaneously recognised and attenuated because it is anticipated, routinised, or cognitively aligned with prior expectations [14,18]. Rather than being either detected or overlooked, discrimination may be recognised in ways that minimise its disruptive implications. In this regard, gendered priors do not merely influence the content of social judgements; rather they shape the inferential processes through which social reality is constructed as meaningful.

Building on this framework, this paper introduces the concept of predictive recognition of discrimination. In this paper, recognition is approached as an inferential process coming from the dynamic interaction between situated social input, pre-existing gendered expectations, and ongoing interpretative updating. Within this framework, when discriminatory interactions are consistent with internalised social priors, they would be expected to generate limited interpretative disruption or, in Clark’s [10] terms, they produce minimal prediction error. In such cases, they are processed as expected and socially coherent rather than as events warranting critical re-evaluation. Discrimination can therefore become cognitively and socially understandable, making it less likely to be recognised or identified as a problem and, as a result, subjected to in-depth reflective analysis.

### 1.2. The Recognition–Normalisation Paradox

Building on this theoretical perspective, this paper proposes the concept of the *recognition–normalisation paradox*. Existing explanations for the persistence of gender discrimination have frequently appealed limited awareness or insufficient consciousness of inequality as the primary operative mechanism [5,9]. However, the interviews analysed in this study reveal a more complex and theoretically rich dynamic: individuals often acknowledge gender discrimination, but at the same time downplay its significance through interpretive reinterpretation. Participants may acknowledge the unfairness of an interaction while concurrently reframing it as ordinary and expectable, contextualising it within a generational or environmental logic, or minimising its weight through diminishing frames. This pattern is consistent with psychological research on attributional ambiguity [7,19] and on the motivated management of discriminatory experiences [8,20], which has demonstrated that the perceived social, relational, and psychological costs of identification frequently lead individuals to overlook or weaken experiences that might otherwise be labelled as discriminatory. Recognition, therefore, is neither binary nor established. It is evaluated, negotiated, contextually situated, and subject to ongoing revision. Individuals may inhabit ambiguous and shifting positions between full acknowledgement and complete denial, deploying different interpretative frames across contexts and interlocutors. This dynamic aspect of recognition has not been sufficiently explored in previous studies, which have tended to view awareness as a binary variable—either present or absent—rather than as a complex inferential process.

From this perspective, the recognition-normalisation paradox can be formally defined as the simultaneous acknowledgment of gender discrimination and its discursive downplaying through interpretive frameworks that make it socially understandable, expected, and unavoidable. From a predictive processing perspective [10,14], this paradox can be interpreted as arising from the same inferential system that enables recognition while also constraining it. Gendered priors do not only guide attention toward potentially discriminatory cues but, also, they simultaneously stabilise interpretation by aligning such cues with established expectations. When discriminatory patterns are cognitively predicted and socially coherent, they generate limited prediction error and therefore fail to trigger sustained critical disruption. The result is a form of recognition that is epistemically genuine yet practically passive: a knowing awareness that does not translate into critical re-evaluation or behavioural response.

Despite the relevance of adjacent theoretical frameworks, the recognition–normalisation paradox proposed in this study contributes to the literature in three ways. First, existing approaches to gender discrimination have often framed normalisation as a consequence of lack of awareness, perceptual invisibility, or ideological internalisation (e.g., system justification or benevolent sexism) [5,9]. Within these perspectives, normalisation tends to be understood as the absence or failure of recognition. By contrast, the present framework conceptualises normalisation as occurring in the presence of recognition. The empirical patterns examined in this study suggest that individuals may explicitly acknowledge discrimination while simultaneously attenuating its significance [7,8], indicating that recognition and normalisation are not mutually exclusive but co-constitutive processes. Second, while previous research has documented interpretative ambiguity and motivated reasoning in responses to discrimination, it has generally treated recognition as a relatively discrete or threshold-based variable (i.e., either present or absent) [7,8,19]. This study proposes instead that recognition is a graded and dynamically negotiated process, embedded within broader interpretative frameworks that can both enable acknowledgment and constrain its consequences. Third, the concept of the recognition–normalisation paradox opens a new line of theoretical integration with predictive social cognition [10,14]. Rather than treating normalisation as a purely ideological or discursive phenomenon, it can be interpreted as part of a broader inferential process through which individuals align social experience with pre-existing expectations. In this sense, the paradox does not merely describe a pattern but provides a conceptual bridge between qualitative research on discrimination and models of predictive cognition. Taken together, these contributions position the recognition–normalisation paradox as a novel conceptual tool for understanding how awareness of discrimination is maintained while its disruptive implications are simultaneously contained.

### 1.3. Comparative Perspective: Italy and Lithuania

The comparative dimension of this study is not designed to identify superficial cultural differences but to examine how distinct socio-historical contexts shape the inferential frameworks (i.e., the gendered priors) through which gender discrimination is experienced and interpreted. Italy and Lithuania represent distinct trajectories with respect to family structures, gender norms, labour market organisation, and the historical transformations of gender relations, offering a theoretically productive basis for cross-national inquiry.

Italy is characterised by a familistic welfare regime and persistently traditional gender norms, with comparatively low female labour market participation, strong occupational segregation, and a cultural context in which the “male breadwinner model” retains normative salience despite significant regional and generational variation [21,22]. By contrast, Lithuania experienced a specific trajectory under state socialism that formally incorporated women into the labour force and public life, yet simultaneously maintained or reconstructed traditional domestic expectations, a configuration that produced a distinctive dual-burden dynamic and a particular mode of gender ambivalence [23,24].

These contrasting contexts are likely to give rise to differently configured gendered priors, which in turn influence how discrimination is recognised, how it is interpreted, and how it is normalised or contested in everyday experience. Critically, these contextual differences are not just background variables; they establish key features of the interpretative process itself, the socio-historical sediment from which gendered priors are formed and reproduced. The comparative design therefore provides an opportunity to examine the recognition–normalisation paradox as it operates across distinct normative and historical configurations, highlighting both its structural invariants and its context-specific modulations. Italy and Lithuania were selected as analytically contrasting contexts rather than as representative cases. While both are European countries, they differ in socio-cultural, historical, and gender regime trajectories, providing a useful basis for exploring how similar patterns may be configured differently across contexts [25,26]. The aim of this comparison is therefore not to generalise at the national level, but to use contextual variation as an analytical resource for identifying variation in interpretative patterns.

### 1.4. Research Aim

This paper addresses the following research question: How do individuals recognise, interpret, and normalise gender discrimination in everyday social experience across Italy and Lithuania? To answer this question, the study draws on semi-structured interviews conducted with participants in both contexts, analysing the narrative repertoires through which discrimination is recognised, the interpretative mechanisms through which it is attenuated, and the ways in which these processes vary across socio-cultural contexts and participant experiences.

## 2. Materials and Methods

### 2.1. Research Design and Epistemological Positioning

The present study adopts a qualitative research design grounded in an interpretivist epistemological framework. This orientation reflects the theoretical commitments developed in the previous section: if discriminatory experience is constitutively interpretative, then the appropriate methodological approach is one that privileges the investigation of meaning-making processes as they unfold within participants’ own accounts and discursive practices [27,28].

The study employs semi-structured interviews as its primary instrument of data collection. Semi-structured interviews offer the dual advantage of providing sufficient thematic structure to ensure cross-case comparability, essential for the comparative dimension of the study, while preserving the flexibility necessary to allow participants to articulate their experiences in their own conceptual terms, to revise and qualify their accounts, and to express the ambivalence and complexity that the recognition–normalisation paradox characteristically entails. The interview setting provides a degree of reflexive distance from the immediate contexts of discriminatory experience, allowing for forms of retrospective interpretation that may be analytically distinct from the inferential processes at work in the moment and, at the same time, bring them to light [29]. The approach is explicitly comparative, drawing on data collected in two national contexts, Italy and Lithuania, selected based on theoretical criteria rather than purely representative ones. As illustrated in Section 1.4, these two contexts represent contrasting socio-historical configurations of gender relations and regulatory frameworks, making them rich environments for examining how differently configured gendered social assumptions shape the functioning of the recognition–normalisation paradox. The comparative logic is therefore analytical rather than enumerative: the aim is not to produce generalisable national profiles but to exploit contextual variation as a resource for theoretical elaboration [28].

### 2.2. Sampling Strategy and Participant Recruitment

Participants were recruited through a purposive sampling strategy designed to maximise variation across dimensions theoretically relevant to the research questions: gender identity, age group, occupational domain, and educational background [28,29]. Purposive sampling was selected in preference to probability-based approaches on the grounds that the analytic aim of the study, the identification and elaboration of interpretative patterns and their contextual variation, requires diversity of experience rather than representational adequacy in a statistical sense.

The sample comprises 25 participants in total: 14 recruited in Italy and 11 in Lithuania. In both national contexts, recruitment targeted individuals across the gender spectrum, including women, men, and individuals identifying as non-binary or gender non-conforming, to examine whether and how gender identity shapes the configuration of gendered social priors and the interpretative dynamics through which discriminatory experience is processed. Inclusion of participants across the gender spectrum also reflects the theoretical proposition that gendered priors are not exclusively or uniformly held by any single gender category but are differentially configured and activated across gender positions. Participants were recruited through institutional contacts, professional networks and snowball referral, with explicit attention on avoiding homogeneity of occupational or educational context. The age range of the sample extends from 19 to 50, with deliberate inclusion of participants across generational cohorts to capture potential variation in the normative landscapes within which gendered social priors have been formed. Recruitment continued until thematic saturation was approached within each national sub-sample, the point at which additional interviews ceased to yield substantially new interpretative repertoires or attenuation mechanisms [30,31]. Sample characteristics are summarised in Table 1.

### 2.3. Interview Procedure and Thematic Guide

Interviews were conducted online in two different languages, Italian or English, according to participants preference and language proficiency. Participants in the Lithuanian subsample selected the interview language based on their fluency, typically opting for English and, in some cases, Italian. Although interviews were not conducted in Lithuanian due to the researcher’s lack of proficiency in the language, all participants reported a high level of competence in the chosen language. It is acknowledged that conducting interviews in a non-native language may have influenced the expressive richness of some accounts. In a limited number of cases, responses appeared more concise or less elaborated; however, this pattern was not systematic and also reflected the absence of relevant experiences (e.g., participants reporting that no such episodes had occurred). Each interview was audio-recorded with participant’s informed consent and transcribed verbatim. The interview guide was structured around three thematic areas, related to everyday environments: family, social and work/educational. For each area participants were asked to reflect about gender-based discrimination, through episodes, examples, behaviours or beliefs that they might have face directly or as bystanders and to think about their own emotion, if not elicited from the free narration of the episodes. Finally, participants were also asked to reflect on their own discriminating behaviours or beliefs. Interview duration ranged from approximately 25 to 58 min, with a mean of 30 min.

Interviews were analysed in their original language (Italian or English). For reporting purposes, extracts were translated into English where necessary, while the original wording was preserved in data repository to ensure transparency and interpretative accuracy.

### 2.4. Data Analysis

Data analysis was conducted using a qualitative–quantitative integrated approach, operationalised through ATLAS.ti Version 26.0.1 [32]. This methodological choice reflects the dual analytical objectives of the study: first, to identify and describe the qualitative repertoires through which recognition and normalisation operate within individual accounts; and second, to examine the distributional properties of these repertoires across participants, contexts, and demographic categories in a manner that permits systematic cross-case comparison.

The analytical process proceeded in three principal phases. In the first phase, an initial coding framework was developed inductively from a subset of 10 transcripts 5 Italian and 5 Lithuanian, selected to maximise contextual and demographic variation. Open coding was applied to these transcripts without a predetermined categorical scheme, generating a preliminary inventory of codes. This phase drew on the reflexive thematic analysis framework proposed by Braun and Clarke [33], adapted to foreground the interpretative and inferential dimensions of participants’ accounts rather than their purely experiential content. The unit of analysis for coding consisted of manually identified text segments corresponding to discrete semantic units (i.e., a phrase or a coherent conceptual fragment). Segments were defined interpretatively as portions of text conveying a single meaning, theme or example and were delimited when a shift in topic, referent, or narrative focus occurred. As such, coding units did not follow fixed syntactic boundaries but were constructed to preserve the meaningful structure of participants’ accounts. Coding was conducted by the author through an iterative and reflexive process. To enhance the robustness of the analytical procedure, the coding framework was reapplied to the dataset after a three-month interval, allowing for internal consistency checking and refinement of code definitions.

In the second phase, the preliminary coding framework was refined through systematic application to the full corpus of 25 transcripts within ATLAS.ti. Codes were revised, merged, and differentiated iteratively in response to the empirical material, in accordance with the principles of constant comparison [34]. The final codebook comprised 41 primary codes, organised into 5 thematic families, the structure of which is presented in Table 2.

In the third phase, ATLAS.ti quantitative output functions were employed to examine the frequency and co-occurrence patterns of codes across the corpus. Code frequency analysis enabled identification of the most prevalent recognitive and attenuative repertoires within each national sub-sample and across gender identity categories. Co-occurrence analysis was used to examine the structural relationships between recognitive and attenuative codes within individual transcript segments, providing empirical grounding for the observation that recognition and normalisation co-occur within the same discursive segments rather than constituting temporally or analytically discrete processes. This integrated approach draws on the methodological logic of what Teddlie and Tashakkori [35] describe as concurrent embedded design: qualitative analysis constitutes the primary analytical framework, while quantitative operations serve to enhance precision, facilitate cross-case comparison, and render distributional patterns visible that may not be readily apparent through purely interpretative means [27]. The quantitative strand does not test hypotheses or produce inferential statistics; it rather functions as a systematic procedure for the documentation and communication of analytical findings that is transparent and accountable in its operations [36].

### 2.5. Reflexivity and Quality Criteria

In accordance with the interpretivist commitments of the study, quality is assessed not through the criteria of reliability and validity appropriate to positivist research design, but through the criteria of credibility, transferability, dependability, and confirmability. Credibility was pursued through engagement with the corpus and use of negative case analysis to identify instances that challenged or complicated the emerging theoretical framework. Transferability is supported by the thick description of context, sample characteristics, and analytical procedures provided in this section, enabling readers to assess the applicability of the findings to other settings. Dependability and confirmability are addressed through maintenance of a methodological audit trail within ATLAS.ti, documenting the evolution of the coding framework and the analytical decisions made at each stage of the process.

The use of analytic memos also supported reflexive engagement with the data, allowing the researcher to critically monitor the interpretative process and to minimise the risk of imposing theoretical assumptions onto participants’ accounts.

Given the theoretical centrality of the concept of gendered social priors to the analytical framework, particular attention was given to the risk of projecting inferential mechanisms onto participants’ accounts rather than identifying them as empirically grounded features of the data. The analytical process was accordingly structured to ensure that the theoretical vocabulary of predictive social cognition was applied in interpretation rather than in coding; that is, that the primary codes remained as close as possible to the language and structure of participants’ own accounts, with theoretical elaboration reserved for the subsequent interpretative layer.

### 2.6. Ethical Information

The study received ethical approval from Joint Committee on Research Ethics, Faculty of Philosophy, Vilnius University (Approval reference: No. (1.13 E) 250000-KT-169 of 9 October 2024). All participants provided written informed consent prior to interview, having been informed of the voluntary nature of their participation, their right to withdraw at any point without consequence, and the procedures through which data would be stored, anonymised, and reported. Confidentiality was protected through systematic anonymisation of all identifying information in transcripts and analytical outputs; participants are identified throughout this paper by alphanumeric codes indicating national context and interview number (e.g., IT-07, LT-14). All data were stored in encrypted repositories accessible only to members of the research team, in compliance with applicable data protection regulations. Given the sensitive character of the subject matter, particular care was taken to ensure that the interview setting was experienced as safe and non-coercive. The interviewers was a trained psychologist who also had a list of support services, if needed after the interview.

## 3. Results

Results reported in this section are presented as discoursive and interpretative patterns emerging from participants’ narratives. Within the text, some excerpts from the interviews have been included; however, whilst these excerpts are representative, they do not cover all the data collected. For this reason, please refer to the dataset published on Zenodo, which is linked in the notes at the end of the paper. In the results, Groundedness (Gr) refers to the number of coded segments associated with a given code across the dataset. As multiple codes could be applied to the same segment, groundedness values should be interpreted as indicators of code density rather than mutually exclusive frequencies.

### 3.1. Normalisation as Core Interpretative Focus

Across the full dataset (*n* = 638 coded segments), the co-occurrence analysis reveals a highly structured configuration of interpretative patterns in which gender discrimination is rarely articulated in isolation. Instead, discriminatory episodes are systematically embedded within interpretative frameworks that attenuate their disruptive potential. The code Normalisation (Gr = 42) emerges as one of the most densely connected nodes in the entire network, functioning as a central hub through which multiple interpretative moves converge. Specifically, normalisation co-occurs consistently with codes capturing socially shared expectations (Social norms, Gr = 38), entrenched representational schemas (Gender Stereotypes, Gr = 61), internalised devaluation (Internalised misogyny, Gr = 62), and perceptual down-weighting (Indifference/underestimation, Gr = 25). This clustering indicates that the attenuation of gender discrimination does not operate as a singular or isolated interpretative move, but rather as a layered process embedded within and reproduced through the socially shared normative structures. Normalisation is not exclusively associated with the absence of recognition. It also co-occurs with codes indicating explicit awareness of inequality, including Women status (Gr = 47) and Gender disparity (Gr = 19). This empirical pattern constitutes initial evidence for the recognition–normalisation paradox: the same narrative sequences frequently contain both the identification and the simultaneous attenuation of discriminatory content.

### 3.2. The Interpretative Ambiguity

Across the interviews, participants frequently identify situations that are classifiable as instances of gender discrimination, including workplace inequality, gendered role expectations, and everyday sexist interactions. However, such recognition is rarely categorical or stable. Co-occurrence patterns show that codes capturing Workplace Discrimination (Gr = 17), Gender segregation (Gr = 18), and Mental load (Gr = 13) are consistently linked with interpretative mechanisms that weaken their evaluative clarity.

Discriminatory episodes are recurrently reframed as context-dependent, attributed, for instance, to generational differences (Generational impact, Gr = 16), reinterpreted as unintentional or unconscious in nature, or embedded within routine practices such as the domestic division of labour and care work. These patterns collectively indicate that recognising discrimination requires active interpretative work under conditions of persistent ambiguity: episodes are rarely experienced as unequivocal violations, but rather as socially embedded interactions that resist straightforward classification.

### 3.3. Patterns of Attenuation: From Recognition to Normalisation

The co-occurrence analysis allows the identification of several recurrent mechanisms through which initial recognition is subsequently attenuated. These mechanisms are analytically distinct, though they frequently operate in combination within the same narrative sequence.

#### 3.3.1. Trivialisation Through Irony

A prominent co-occurrence pattern links Normalisation with Irony (Gr = 24). Discriminatory content is frequently reframed as humour, enabling participants to simultaneously acknowledge problematic elements and neutralise their moral weight. By relocating the episode into the culturally legitimised domain of joking practices, this mechanism reduces the perceived severity of the interaction while preserving its social acceptability.

*“We’ve sort of got used to the way our male friends joke around… even when they make jokes that aren’t exactly politically correct, it’s become a bit of a given because we know that’s just how they are”*

#### 3.3.2. Diffusion and Displacement of Responsibility

Codes associated with moral disengagement—Diffusion of Responsibility (Gr = 7) and Attribution of Blame (Gr = 7)—co-occur with both discrimination-related episodes and normalisation. These patterns reveal a consistent interpretative strategy whereby individual accountability is redistributed across the community, victims are partially implicated in the interaction, and responsibility is reduced to the point of incontestability. These patterns are associated with accounts in which discrimination is presented as socially intelligible and expected, rather than individually accountable.

*“I see others going along with it and laughing at the joke… so the incident remains somewhat unpunished, as if we all approved of it”*

#### 3.3.3. Perceptual Down-Weighting and Indifference

The relatively high groundedness of Indifference/underestimation (Gr = 25) and its systematic co-occurrence with Normalisation identify a qualitatively distinct attenuation mechanism: the reduction in perceived salience rather than explicit justification. In these instances, discrimination is acknowledged but framed as minor or inconsequential, described as not warranting a reaction, and put as background noise within everyday social interaction. This pattern is characterised by a reduction in the perceived salience of the episode rather than explicit evaluative judgement, making it analytically distinct from mechanisms involving active reframing.

#### 3.3.4. Internalisation of Gendered Expectations

The strongest co-occurrence patterns in the dataset involve Internalised Misogyny (Gr = 62) and Gender Stereotypes (Gr = 61), both tightly linked with Normalisation. Participants frequently reproduce gendered expectations, describe them as natural or historically rooted, and align their own interpretations with such expectations even when acknowledging their perceived unfairness. These patterns suggest that the interpretative process is deeply structured by internalised social schemas that are reproduced within participants’ accounts and are not always explicitly problematised.

*“It wasn’t as if I wanted to denigrate her… it just seemed normal at the time”*

### 3.4. Social Norms as Mediating Infrastructure

Social norms (Gr = 38) emerge as a central interpretative category linking discriminatory episodes and their normalisation within participants’ accounts. Co-occurrence patterns reveal that discriminatory behaviours are consistently framed as conforming to shared expectations, with participants invoking normative consensus to justify or attenuate their significance. Social routines (including family role distributions, workplace hierarchies, and peer interaction scripts) serve as interpretative anchors that make discrimination comprehensible but also implicitly appropriate. These findings suggest that normalisation is not just an individual cognitive strategy but is socially supported through shared normative frameworks. Social norms are conceptualised as a mediating infrastructure because they do not directly produce discriminatory behaviour, but provide the shared interpretative framework through which discriminatory experiences become understandable, acceptable, and open to attenuation.

*“In the end, these things are not done with the intention to discriminate… they have simply become ingrained over time”*

### 3.5. Recognition–Normalisation Configurations

Recognition and normalisation appear to be systematically tangled within the same narrative sequences. In particular, three configurations recurring emerge from the co-occurrence analysis. The first involves participants explicitly recognising a discriminatory episode but immediately qualifying it as generational, unintentional, or context-specific, thereby preserving their identification of the phenomenon while simultaneously restricting its generalisability and moral urgency.

*“It makes me feel that it’s unfair… but it also seems so entrenched that I feel I can’t really do anything about it”*

The second configuration is evidenced by the co-occurrence of Absence/non-discrimination (Gr = 27) with multiple discrimination-related codes. Participants deny the existence of discrimination while providing narrative examples that correspond precisely to it, producing an interpretative tension that is never explicitly resolved. The third configuration is associated with the code Acceptance (Gr = 12). Here, discrimination is recognised and its structural features acknowledged, but it is simultaneously framed as inevitable, making it unchangeable from individual agency and, sometimes, also collective challenge.

*“I’ve been expected to do certain things because of my gender… and I usually comply with them”*

### 3.6. Cross-National Variation in Normalisation Configurations

Italian interviews account for approximately 65% of coded material (*n* = 413), with Lithuanian interviews representing the remaining 35% (*n* = 225). Despite this distributional asymmetry, the recognition–normalisation paradox is observable in both national datasets. The comparison presented here is analytical rather than representative: it is intended to highlight differences in the configuration of interpretative patterns within this sample, rather than to characterise national cultures. Nonetheless, the two datasets diverge substantially in the specific configurations through which normalisation is accomplished. Percentages reported in this section refer to the distribution of coded segments across national subsamples, rather than to the prevalence of codes within each group or the proportion of participants expressing them. As such, they should be interpreted as comparative indicators of distribution within the analytic corpus, considering the overall imbalance in dataset size.

#### 3.6.1. Italy: Normalisation Through Routinised Social Expectations

The code Normalisation is more strongly represented in the Italian data (73.8% of occurrences), and this asymmetry is mirrored by closely associated codes: Social norms (73.7% Italian), Male role (71.4% Italian), and Mental load (76.9% Italian). This pattern suggests that, within this dataset, normalisation in the Italian context is predominantly associated with everyday social structures and routinised gender expectations. Italian interviews further show a strong clustering of normalisation mechanisms around internalised norms and perceived constraints on agency: Internalised misogyny (59.7% Italian), Perceived responsibility (87.5% Italian), and Inability to react (87.5% Italian). Additionally, certain codes are exclusively present in the Italian data, such as Diffusion of responsibility and Paternity, reinforcing the centrality of collective and familial structures in shaping interpretative frameworks.

*“I think the most evident cases of normalisation are the jokes… simply not recognising the space they occupy”*

Taken together, these patterns suggest that, within the Italian interviews, discrimination is often framed as socially expected, and individuals perceive limited agency to contest it; interpretative attenuation is accomplished primarily through internalised norms and relational obligations rather than through explicit discursive strategies. Qualitatively, the dominant Italian configurations involve recognition followed by acceptance, awareness of inequality combined with a perceived impossibility of action, and a strong embedding of discriminatory experience within family and relational expectations. Discrimination is thus frequently processed as socially coherent and structurally embedded within existing relational expectations, rather than as a contingent or contestable event.

#### 3.6.2. Lithuania: Normalisation Through Relativisation and Discursive Distancing

Recognition of gender inequality is present in the Lithuanian data to a comparable extent, though it is more explicitly tied to structural and institutional domains. Codes capturing structural discrimination: Gender disparity (57.9% Lithuanian) and Gender segregation (61.1% Lithuanian) are proportionally more prominent in Lithuanian interviews, while experiential and relational articulations of inequality (Women status: 74.5% Italian) predominate in the Italian data. Normalisation within the Lithuanian interviews is accomplished through a qualitatively different configuration. Several attenuation-related codes are proportionally more prominent in the Lithuanian data: Absence/non-discrimination (59.3% Lithuanian), Moral justification (80% Lithuanian), and Paternalism (60% Lithuanian).

*“It’s normal, but… why can’t I express myself or dress as I want?”*

Rather than grounding normalisation in the routines of everyday life, Lithuanian participants more frequently employ discursive strategies of negation, relativisation, and contextualisation, framing discrimination as exceptional, situationally determined, or historically conditioned. The dominant Lithuanian configurations involve recognition combined with denial, structural acknowledgement paired with individual distancing, and normative relativisation. This pattern in the dataset suggests that normalisation in Lithuania operates more at the level of explicit discursive framing than through the embodied reproduction of social routines.

### 3.7. Shared Mechanisms Across Contexts

Despite the cross-national differences documented above, several attenuation mechanisms appear consistently across both datasets. Irony exhibits a relatively balanced distribution (62.5% Italian; 37.5% Lithuanian), indicating that trivialisation through humour operates transversally as a mechanism that permits recognition while simultaneously neutralising its evaluative weight. Indifference/underestimation is similarly distributed across both contexts (64% Italian; 36% Lithuanian), pointing to a shared process of reducing the salience of discriminatory experiences and framing them as non-critical.

Most significantly, both Gender stereotypes (55.7% Italian/44.3% Lithuanian) and Internalised misogyny (59.7% Italian/40.3% Lithuanian) are highly frequent across both national contexts. The relatively balanced distribution of these codes suggests that, within the dataset, internalised gender expectations constitute a shared interpretative infrastructure, a set of shared normative schemas that appear to structure how discriminatory experience are interpreted within participants’ accounts, regardless of national context, even as the surface configurations of normalisation differ.

Taken together, the co-occurrence analysis and the cross-national comparison converge on a coherent empirical picture. Recognition of gender discrimination is present in both contexts and at both levels of analysis. Normalisation processes are systematic rather than incidental: they involve multiple, interacting mechanisms that are socially scaffolded and deeply embedded in shared normative frameworks. The attenuation mechanisms identified (e.g., trivialisation, diffusion of responsibility, perceptual down-weighting, and internalisation) are not mutually exclusive but tend to operate in layered combinations. Cross-nationally, these mechanisms are configured differently: normalisation in Italy is more closely tied to the routinised reproduction of everyday social expectations and perceived constraints on individual agency, while in Lithuania it operates more through explicit discursive relativisation and contextual distancing. Nonetheless, in both cases, gender discrimination is processed through interpretative frameworks that render it socially coherent, and less likely to be critically contested within participants’ account.

## 4. Discussion

The findings reported in this study provide strong empirical support at the level of narrative and interpretative patterns as a structuring feature of how individuals relate to gender discrimination in everyday experience. Across both national contexts, participants demonstrated a consistent and analytically significant pattern: discrimination was acknowledged, named, and at times explicitly evaluated as unjust, yet this acknowledgement was systematically accompanied by interpretative moves that attenuated its significance, contained its implications, and precluded its translation into sustained critical contestation. These two processes, recognition and normalisation, do not operate as separate, and sequential stages, they co-exist within the same moment. Participants’ accounts reveal a characteristic structure in which a discriminatory episode is named and immediately recontextualised, minimised, or otherwise rendered less consequential. Recognition and minimisation are, in this sense, not opposed but intertwined: the former frequently appears to enable the latter, providing the epistemic ground from which it develops.

This pattern is consistent with, but also advances beyond, prior psychological research on the reporting and contestation of discriminatory experiences. Swim and Hyers [20] documented a widespread tendency among women to refrain from overt responses to sexist remarks, even when these were clearly identified as offensive. Stangor et al. [37] similarly observed significant discrepancies between private recognition and public reporting of discrimination. The present findings extend this literature by demonstrating that the gap between recognition and contestation is not merely behavioural, but interpretative: it is constituted within the very cognitive and discursive frameworks through which the experience is processed. The data support a reconceptualization of normalisation not as the absence of recognition, but as a specific mode of handling it [38], where awareness is simultaneously sustained and contained.

A dominant interpretive framework in research on the persistence of gender discrimination considers normalisation as a consequence of a lack of awareness of discriminatory practices, particularly the more subtle ones, and of the internalisation of ideologies that obscure the gendered aspects of social structures [5,9]: individuals fail to challenge discrimination because they fail to recognise it as such.

The present findings challenge this interpretative framework. Participants in this study frequently demonstrated an explicit and articulate capacity to recognise and identify inequality. The problem, as the data reveal, is not that discrimination goes unnoticed, rather that noticing does not reliably translate into critical challenge. Participants who recognised a discriminatory dynamic often simultaneously deployed interpretative frames that attenuated its significance: while recognition was present; its transformative potential was systematically constrained. The distinction between recognition and critical disruption is theoretically consequential. Normalisation operates not as a cognitive absence but as a specific mode of cognitive and discursive processing. From this perspective, the relevant question is not whether individuals recognise discrimination, but rather what happens to that recognition within existing interpretative frameworks. This reorientation connects the present findings to a broader literature on motivated cognition and system justification. Jost and Hunyady [39] have argued that individuals are psychologically motivated to perceive the social systems they inhabit as fair and legitimate, even when these systems operate to their disadvantage. Crosby [40] documented the denial of personal discrimination in contexts where group-level discrimination is simultaneously acknowledged, which was attributed to the cognitive and emotional costs of perceiving oneself as a victim. The present framework extends these insights by specifying the inferential mechanisms through which such motivated processing operates, drawing on predictive processing theory as a framework to interpret the possible cognitive dynamics that may sustain recognition without disruption.

In this regard, the recognition–normalisation paradox contributes to the literature by reframing the relationship between recognition and normalisation: rather than treating these as oppositional processes, the present analysis highlights their systematic co-occurrence within the same interpretative sequences, thereby challenging models that equate normalisation with absence of awareness.

The analysis did not systematically differentiate patterns by participants’ gender, as the focus of the study was on shared interpretative mechanisms rather than group-level differences. While gendered variations may be relevant, they fall outside the scope of the present analysis and represent a potential direction for future research.

### 4.1. The Role of Gendered Social Priors in Predictive Recognition

The theoretical framework developed in this paper proposes that the recognition–normalisation paradox can be interpreted through the lens of predictive social cognition. Drawing on Clark’s [10,12,13] description of the predictive brain and Hinton’s [14] analysis of culturally rooted reasoning patterns, the concept of predictive recognition of discrimination argues that the interpretative processing of discriminatory experience is largely shaped pre-existing expectations through which social interactions are usually organised and evaluated. From a theoretical perspective, the following inferential sequence can be proposed as a plausible interpretative model: gendered social assumptions (e.g., internalised expectations regarding gender roles) serve as the predetermined predictive models through which individuals interpret incoming social information; when a potentially discriminatory interaction occurs, the extent to which it generates critical recognition depends substantially on whether it confirms or violates these assumptions [15]. When an interaction is consistent with established gender expectations (e.g., a promotion denied for reasons implicitly linked to gender, or a domestic responsibility assigned without explicit negotiation) it generates a limited prediction error. According to Clark’s [10] definition, this is an expected outcome, meaning an event that the predictive system has already modelled and for which the available interpretive resources are already aligned, therefore assimilated into existing frameworks rather than triggering their adjustment.

This perspective helps to interpret a central feature of the empirical findings: the frequency with which participants described discriminatory incidents as normal or socially accepted. These formulations are not expressions of indifference or a lack of awareness, but rather the articulation of an experience that has been processed as a confirmation, rather than a violation, of prior expectations. As Hinton [14] argued, culturally transmitted schemas operate as inferential default settings that structure social perception prior to deliberate evaluation. Gender preconceptions, according to this interpretation, do not simply influence the way in which discrimination is evaluated after it has been identified, but shape the very process of identification, determining whether an interaction is recorded as an anomaly that requires explanation or as a routine example of an already familiar pattern. This theoretical explanation also clarifies why discrimination that is more pronounced or more contextually incongruent tends to elicit more intense recognition responses, a pattern that is partially observable in the data. When an interaction violates pre-existing gender expectations rather than confirming them, it produces a greater prediction error, which, within Clark’s [10] theoretical framework, triggers a more prolonged inferential update and, potentially, a critical re-evaluation. Predictive processing theory thus offers a well-founded explanation for the gradual nature of recognition revealed by the data: the intensity of recognition varies, at least in part, according to the extent to which an experience disrupts, rather than confirms, established inferential expectations.

### 4.2. Attenuation Mechanisms as Prediction-Error Management

A further aspect to be discussed is the reinterpretation of specific attenuation mechanisms which, according to empirical data, have emerged as effective strategies for managing prediction errors. These mechanisms can be interpreted, within a predictive framework, as processes that may function to restore consistency between experiences and established schemas, thereby reducing prediction errors without, however, requiring a revision of the underlying gendered priors. Several examples of this can be found in the data. Irony works by recoding a potentially discriminatory interaction as humorous, thereby preventing it from demanding serious evaluative attention. Rather than generating the sustained interpretative engagement that would be necessary to update a gender bias, irony keeps that bias intact by reclassifying the episode as a non-serious matter. By downplaying experiences, participants reduce the significance of an interaction, thereby preventing the triggering of a re-evaluation. Contextualising and explaining episodes (e.g., generational factors) helps to integrate the experience into existing causal narratives, aligning it with pre-existing schemas and avoiding structural re-evaluation [16]. These mechanisms are consistent with current psychological theories on coping with discrimination and represent an extension of them [41]. Major and Schmader [19] described a range of strategies through which individuals cope with the psychological costs of discrimination, including denial, reattribution, and social comparison. Lazarus’s [42] cognitive appraisal model identifies reframing and contextualisation as functional responses to potentially threatening experiences. The contribution of the present analysis lies in situating these mechanisms within a predictive processing framework: instead of considering them strategies of psychological self-protection, they can be analytically interpreted as inferential operations that function, at a theoretical level, to maintain stability within existing interpretative frameworks. These mechanisms do not eliminate recognition but rather contain it: awareness of discrimination is preserved, whilst its capacity to serve as a basis for critical re-evaluation is systematically neutralised. According to this theoretical framework, this configuration may represent a relevant pattern in how gender inequality is perpetuated at the level of everyday cognition and social interaction.

Recognition is therefore not a threshold variable, which can be present or absent, but rather a graded, negotiable and contextually situated process. This description is substantially supported by empirical evidence, which rarely portrays recognition as a simple, stable state of awareness; rather, it is more commonly characterised by a distinctive pattern of contextual revision, reflecting the fundamentally ambivalent nature of recognition as it is experienced. This gradual quality has several analytically significant dimensions. First, recognition varies in intensity: the same participant may describe one episode as clearly discriminatory and another as ambiguous or irrelevant. Second, recognition varies across contexts: participants may express a more explicit recognition of discrimination in interview settings, where reflective distance is structurally available, compared to the very moments when the event occurs. Finally, recognition is subject to retrospective revision: accounts that initially downplay a discriminatory episode may be revisited and recalibrated, suggesting that the inferential process is ongoing and responsive to conversational cues that activate different a priori configurations.

These dynamics tie into broader issues in the psychology of discrimination concerning the relationship between private experience and public expression. According to Barrett and Swim [7] the assessment of prejudice is a multi-stage process involving both automatic and deliberative components; the outcome of this process is strongly influenced by contextual factors that affect the availability and relevance of various interpretive schemas. The results of this study support and expand this view by demonstrating that the instability of recognition is not incidental to the phenomenon but is constitutive of it. It is precisely because recognition is processual and context-sensitive that it can simultaneously serve as the basis for acknowledgment and as a condition of possibility for mitigation.

This insight has implications for how the relationship between recognition and social change should be theorised. If recognition were binary and stable, the central challenge would be to raise awareness. If recognition is, as this analysis proposes, gradual and subject to continuous inferential management, the challenge is structurally more complex: it requires paying attention to the interpretive frameworks within which recognition is processed, not simply to the occurrence of recognition itself.

### 4.3. Cross National Variation as Social Priors’ Configuration Variation

The comparative dimension of this study reveals significant cross-national variation in how the recognition-normalisation paradox operates; according to the present theoretical framework, this variation reflects not only cultural differences but also structurally distinct configurations of gender-related social assumptions. Italy and Lithuania represent contrasting socio-historical trajectories that have generated different normative frameworks for the organisation of gender relations, and these differences are evident in the interpretive strategies through which participants in each context process and mitigate experiences of discrimination. The cross-national differences discussed here should be interpreted cautiously and in line with the exploratory and analytical nature of the study. The aim is not to provide generalisable descriptions of national contexts, but to use contextual variation as a resource for identifying how different configurations of interpretative patterns may emerge.

In the Italian context, normalisation appears to operate particularly strongly through the activation of relational and family patterns. Discriminatory incidents are often embedded in narratives structured around traditional relational frameworks (e.g., expectations regarding family roles, domestic responsibilities, and intergenerational dynamics) that function as deeply rooted assumptions, lending an apparent naturalness and legitimacy to gender asymmetries. This is consistent with the structural characteristics of the Italian gender regime, characterised by a family-centred welfare model, persistent occupational segregation, and a regulatory landscape in which the domestic organisation of gender retains significant cultural relevance [21]. Within this framework, the discrimination that occurs within relational or family contexts generates a particularly limited prediction error, as it is fully in line with the most deeply rooted gender expectations.

In the Lithuanian context, normalisation most often operates through what might be described as discursive relativisation and contextual distancing: the attribution of discriminatory experiences to specific situational, generational or institutional contexts that contain their implications without assimilating them into a naturalising narrative. This model may reflect a distinctive feature of the post-socialist gender configuration in Lithuania: the formal incorporation of women into the public sphere under state socialism, which produced a superficial norm of gender equality that coexists with the persistence of traditional domestic expectations [23,24]. Within this configuration, gender equality can be invoked as the standard against which specific episodes are measured and found wanting; yet this normative availability does not translate into a sustained critical rupture, as the episodes are simultaneously contextualised as situational deviations from a general norm rather than as expressions of a systemic pattern.

These cross-national differences are theoretically significant for two reasons: on the one hand, they provide empirical evidence to support the argument that gender-related social assumptions are shaped by socio-historical factors rather than being universal; on the other hand, they demonstrate that the recognition–normalisation paradox is not context-specific but contextually shaped: the same fundamental dynamic (recognition without critical interruption) is operationalised through different inferential pathways in different socio-historical contexts. The paradox is structural; its specific form is contextual.

It is important to clarify that the predictive processing framework employed in this study is not empirically tested within the present design. The qualitative data provide access to discursive and interpretative patterns, not to underlying cognitive mechanisms. The proposed framework should therefore be understood as a theoretically grounded interpretative model that offers a possible explanation of the observed patterns rather than a direct account of the cognitive processes involved.

## 5. Conclusions

This article offers three main theoretical contributions regarding gender discrimination, social cognition and the psychology of inequality. Firstly, it introduces and explores the concept of the recognition–normalisation paradox as a generative theoretical framework for understanding the relationship between awareness and acceptance in the context of everyday gender discrimination. If we reconceive normalisation as a specific mode of the cognitive management of recognition, the theoretical framework goes beyond existing explanations that confuse normalisation with invisibility or unawareness. This distinction shifts the analytical focus from the conditions of recognition to those of its containment and provides a more accurate description of the phenomenology of the discriminatory experience as recounted by those who experience it.

Secondly, the paper proposes a shift in the conceptualisation of the normalisation of discrimination in gender studies: rather than interpreting it as a lack of awareness, it regards it as the result of an inferential process. From this perspective, discrimination persists not because of an inability to recognise it, but as a result of a system of gendered social priorities that shapes what is noticed, how it is interpreted, and the likelihood that such an interpretation will trigger critical reflection. This reorientation links the literature on gender discrimination to broader theories of predictive social cognition [10,14] and offers a more parsimonious theoretical interpretation that is consistent with the empirical patterns identified of how discrimination can be simultaneously recognised and perpetuated.

Thirdly, this article highlights the fruitful integration of gender studies and predictive processing theory as complementary analytical frameworks. Applications of predictive processing theory to social cognition have so far focused predominantly on the perceptual and clinical domains [43]; its extension to studies on gender discrimination, however, opens up new theoretical and empirical perspectives. In this context, the literature on gender discrimination offers a field of social experience in which it is possible to observe the mechanisms of predictive inference operating under conditions of normatively structured ambiguity, conditions that are theoretically fruitful for the further development of predictive social cognition as an interpretative tool for the reproduction of cultural patterns through individual inferential practices.

The findings of this study have significant implications both for research and for practices relating to gender equality. From a theoretical and methodological perspective, they suggest that the traditional operationalisation of awareness as a “threshold” variable, typical of survey and experimental studies, risks presenting a distorted picture of the phenomenon of recognising discrimination. If recognition is in fact gradual, context-dependent and subject to continuous inferential revisions, then tools that treat it as a binary category, merely asking whether an experience was recognised as discriminatory or not, prove insufficient [6]. Qualitative and longitudinal approaches, capable of capturing its processual, ambivalent and context-sensitive nature, could offer a more accurate understanding of the ways in which discrimination is experienced and managed in everyday life. At a practical level, the findings suggest that strategies aimed solely at raising awareness of gender discrimination may not, on their own, be sufficient to bring about a genuine challenge to existing social structures. If normalisation does not equate to a lack of recognition, then interventions that promote recognition without altering the interpretative frameworks within which it is processed risk producing the paradox highlighted by the study: greater awareness unaccompanied by greater challenge. Effective interventions should therefore also address the inferential and cultural assumptions through which discrimination is made socially comprehensible and cognitively predictable, rather than treating mere recognition as the ultimate goal.

### Limitations

The study does, however, have certain limitations. Firstly, the research is based on qualitative data obtained through interviews: whilst this methodological approach is suitable for analysing interpretative processes and narrative repertoires, it does not allow for a direct measurement of the cognitive mechanisms hypothesised by the theoretical framework adopted. The proposed predictive processing model therefore remains predominantly theoretical and inferential in nature: it offers a coherent explanation of the patterns that emerged but does not provide direct empirical evidence of the cognitive mechanisms involved. Future research based on experimental paradigms or methodologies capable of monitoring interpretative processes in real time could help to test these hypotheses more directly. Secondly, the comparative scope of the study is necessarily limited: Italy and Lithuania represent two distinct socio-historical contexts, but do not exhaust the variety of gender regimes and normative contexts within which the paradox between recognition and normalisation can manifest itself. The comparative results must therefore be interpreted with caution, whilst the proposed theoretical framework and its grounding in predictive social cognition retain broader validity. Finally, from a methodological perspective, it should be noted that all the interviews were conducted by a female researcher, including those with men; that although the sample was designed to include participants from different age groups, professional backgrounds and gender identities, it inevitably reflects the inherent limitations of qualitative research; that the age range of the sample (19–50 years) does not cover the full adult lifespan and therefore does not allow exploration of discrimination experiences among older adults; and, lastly, that interviews with Lithuanian participants were conducted in English or Italian rather than in Lithuanian. While participants were fluent in the selected language, conducting interviews in a non-native language may have reduced the expressive nuance of some accounts. This limitation should be considered when interpreting cross-national differences, as subtle culturally embedded meanings may not have been fully articulated.

## Figures and Tables

**Table 1:** Sample characteristics by nation and gender.

	Italy			Lithuania			Total
	Women *n* = 8	Men *n* = 5	Non-Binary *n* = 1	Women *n* = 6	Men *n* = 4	Non-Binary *n* = 1	
Age range	24–44	26–41	26	22–50	19–25	25	19–50
Age mean	30.43	35	--	36.4	22	--	31.23
(SD) ^1^	(8.34)	(5.61)		(10.87)	(3)		(8.64)
Total age range	24–44			19–50			
Total age mean	31.85			30.33			
(SD) ^1^	(7.31)			(10.68)			
Total	14			11			25

^1^ Three participants (1 Italy, 2 Lithuania) did not disclose their age.

**Table 2:** Thematic families and codes.

Code Group	Codes
Moral Disengagement	Attribution of blame
	Dehumanisation
	Diffusion of responsibility
	Displacement of responsibility
	Disregard of consequences
	Moral Justification
Psychological impact	Fragility
	Freedom desire
	Frustration
	Inability to react
	Male emotion
	Mental load
	Perceived Responsibility
	Psychological violence
Social Influence	Absence/non-discrimination
	Deconstruction
	Favouritism
	Gender disparity
	Irony
	Male role
	Paternalism
	Personal safety
	Social bubble
Social Normalisation	Acceptance
	Gender stereotypes
	Generational impact
	Indifference/underestimation
	Internalised misogyny
	Normalisation
	Resistance to change
	Social norms
	Women status
Workplace Perspectives	Care job
	Gender pay gap
	Gender segregation
	Glass ceiling
	Paternity
	Paternity leave
	STEM/HASS
	Workplace discrimination
	Workplace pregnancy

## Data Availability

The original data presented in the study are openly available in Zenodo UnveilGBD Community (https://zenodo.org/communities/unveilgbd/,) accessed on 22 May 2026 at the following link: https://doi.org/10.5281/zenodo.20338548.
